# Distinct grey and white matter changes are associated with the phenomenology of visual hallucinations in Lewy Body Disease

**DOI:** 10.1038/s41598-024-65536-w

**Published:** 2024-06-26

**Authors:** Fabrizia D’Antonio, Alice Teghil, Maddalena Boccia, Giulia Bechi Gabrielli, Giovanni Giulietti, Desirée Conti, Antonio Suppa, Andrea Fabbrini, Marco Fiorelli, Francesca Caramia, Giuseppe Bruno, Cecilia Guariglia, Dag Aarsland, Dominic Ffytche

**Affiliations:** 1 Department of Human Neuroscience, Sapienza University of Rome, Viale Dell’università 30, 00185 Rome, Italy; 2 Cognitive and Motor Rehabilitation and Neuroimaging Unit, IRCCS Fondazione Santa Lucia, Rome, Italy; 3 Department of Psychology, Sapienza University of Rome, Rome, Italy; 4 Neuroimaging Laboratory, IRCCS, Fondazione Santa Lucia, Rome, Italy; 5 IRCCS Neuromed Institute, Pozzilli, IS Italy; 6 Department of Old Age Psychiatry, King’s College London, Institute of Psychiatry, Psychology and Neuroscience, IOPPN, London, UK

**Keywords:** Parkinson's disease, Perception

## Abstract

Visual hallucinations in Lewy body disease (LBD) can be differentiated based on phenomenology into minor phenomena (MVH) and complex hallucinations (CVH). MVH include a variety of phenomena, such as illusions, presence and passage hallucinations occurring at early stages of LBD. The neural mechanisms of visual hallucinations are largely unknown. The hodotopic model posits that the hallucination state is due to abnormal activity in specialized visual areas, that occurs in the context of wider network connectivity alterations and that phenomenology of VH, including content and temporal characteristics, may help identify brain regions underpinning these phenomena. Here we investigated both the topological and hodological neural basis of visual hallucinations integrating grey and white matter imaging analyses. We studied LBD patients with VH and age matched healthy controls (HC). VH were assessed using a North-East-Visual-Hallucinations-Interview that captures phenomenological detail. Then we applied voxel-based morphometry and tract based spatial statistics approaches to identify grey and white matter changes. First, we compared LBD patients and HC. We found a reduced grey matter volume and a widespread damage of white tracts in LBD compared to HC. Then we tested the association between CVH and MVH and grey and white matter indices. We found that CVH duration was associated with decreased grey matter volume in the fusiform gyrus suggesting that LBD neurodegeneration-related abnormal activity in this area is responsible for CVH. An unexpected finding was that MVH severity was associated with a greater integrity of white matter tracts, specifically those connecting dorsal, ventral attention networks and visual areas. Our results suggest that networks underlying MVH need to be partly intact and functional for MVH experiences to occur, while CVH occur when cortical areas are damaged. The findings support the hodotopic view and the hypothesis that MVH and CVH relate to different neural mechanisms, with wider implications for the treatment of these symptoms in a clinical context.

## Introduction

Visual hallucinations (VH) are frequent in Lewy Body disease (LBD), and their phenomenology varies widely among patients and according to disease stage. LBD includes Parkinson’s disease (PD), Parkinson dementia (PDD) and Dementia with Lewy bodies (DLB) that share clinical features and neuropathological hallmarks. Visual hallucinations in the LBD spectrum can be differentiated into minor and complex phenomena. Minor phenomena, including passage hallucinations, presence hallucinations and illusions, usually occur in the early stages of disease, and especially in patients with PD^1,2^. Complex visual hallucinations consist of well-formed visions of animals, people or inanimate objects and frequently occur in patients with DLB, constituting a DLB core clinical feature^3^.

Overall, the underlying mechanism of VH is largely unknown. Many models have attempted to explain them, in terms of alterations in different processes, mainly attention^4^, arousal^5^ and the balance between perception and expectations^6^. Recently a synthesis integrating all such views has been proposed delineating cognitive systems relevant to VH^7^. Beyond the cognitive processes, from a localizationist point of view, the models can be divided in those highlighting dysfunction in a specific brain region (a topological approach) and those highlighting an alteration in connectivity between regions (a hodological approach). The hodotopic model can be considered a synthesis of the two approaches. In this model the hallucination state relates to activity in visual areas specialized for the perceptual content of the VH. However, the VH state occurs in the context of structural and functional connectivity alterations among visual areas and other regions, that confer the hallucination trait^8^. It has been hypothesized that minor and complex hallucinations in Lewy body disease might be underpinned by different mechanisms and that VH phenomenology differences might mirror the neuropathological progression in LBD according to Braak stage^1^. This is consistent with the finding that minor visual hallucinations (MVH) are not associated with prominent visual perceptual deficits on neuropsychological testing, contrasting with complex visual hallucinations (CVH) which are associated with visuoperceptual processing, attention and visual abstract reasoning deficits^9^. The absence of visual perceptual deficits makes it unlikely that visual cortical volume loss is associated with MVH given that visual perceptual deficits would be expected to precede cortical volume loss detectable by standard MRI methods. This raises the possibility that MVH might be related to alterations of connectivity within wider networks rather than cortical volume loss in specific regions, in particular, connectivity between subcortical regions (i.e. brain stem) and cortical regions, especially those involved in aspects of visual processing linked to the parietal lobe. Such connectivity changes might reflect axonal and white matter changes in LBD, in which the axonal loss has been found to occur quite early and preceding neuronal loss that may be the result of a dying-back process^10–13^. In contrast, CVH might instead be due to cortical volume loss and dysfunction in ventral visual stream regions with associated neuropsychological deficits. Thus, from the phenomenological perspective of the hodotopic framework, MVH could be interpreted as symptoms related to visual processing network dysfunction, possibly due to axonal and white matter neuropathology in networks playing a modulating role in visual processing, e.g. dorsal and ventral attention networks as well as frontal areas involved in the speed of visual processing. In contrast, while such networks are also involved in CVH, their phenomenology might relate to spontaneous activation of specific cortical areas, with volume loss in these areas reflecting either localised neuropathology or an indirect effect of changes within the wider networks to which they connect. Phenomenology also includes temporal aspects (i.e. the frequency and duration of VH) and the hodotopic model would suggest that hyper- or hypo- connectivity between visual regions and other brain networks may contribute to these phenomenological aspects^9^.

To date, neuroimaging studies on VH in LBD have investigated these phenomena as a whole symptom and have not differentiated between MVH and CVH phenomenology. A review of neuroimaging studies of alterations associated with VH in LBD reported grey matter loss in frontal areas in patients with dementia, and in parietal and occipito-temporal regions in PD without dementia^14^. A recent meta-analysis examining the VBM studies of VH in LBD spectrum reported evidence of lower grey matter volume (GMV) in hallucinating patients in medial superior frontal, and anterior and cingulate cortices, and in the inferior parietal lobule^15^. Studies investigating GMV alterations in PDD patients have reported PDD with VH is associated with reduced GMV in regions that form part of the ventral and dorsal visual streams^16^ or in subcortical regions such as pedunculopontine nucleus and the thalamus^17^. A recent meta-analysis investigating neuroimaging findings in PDD focusing on a network centred on the hippocampus, reported that neuroimaging results in PD patients with VH were heterogeneous, and that neuroimaging abnormalities in PD with hallucinations were part of a common network centred on the lateral geniculate nucleus^18^. In PD, the condition in which MVH most frequently occur, a VBM study showed that MVH are mainly associated with discrete but functionally relevant structural alterations in the dorsal visual stream and functionally related areas involved in visual processing, especially for visuospatial perception^19,20^.

Few studies have investigated structural connectivity alterations associated with VH. In one study VH were found to be associated with structural alterations mainly in the right hemisphere involving attention networks, suggesting a role of attention in generating VH^21^. Recently it has been found that DLB patients with VH had alterations in structural connectivity between the thalamus^22^ and cortical regions, confirming evidence from a previous study that showed a correlation between mean diffusivity within the right thalamic subregion and NPI hallucination score^23^. However, no studies to date have investigated white and grey matter alterations associated with VH phenomenology in DLB. According to the hodotopic approach, and the idea that phenomenology may reveal details of underlying mechanism, the aim of this study was to investigate grey and white matter alterations associated with minor (MVH) and complex VH (CVH) phenomenology, focusing on temporal characteristics. Based on the view that MVH and CVH have a distinct origin^1^, we hypothesized that different structural changes might be associated with MVH and CVH in LBD. Specifically, we hypothesize that CVH are associated with alterations of grey matter in cortical regions, especially those of the ventral visual stream, and consequent alterations of structural connectivity among these visual regions and other networks. MVH might instead be associated with changes in structural connectivity between visual regions and attention networks or within the attention networks themselves but without prominent grey matter alterations. To explore both localised cortical regions and connections between them we used a combination of grey and white matter imaging. We first compared grey matter volume (GMV) and white matter indices (fractional anisotropy (FA) and mean diffusivity (MD) values) in LBD patients with VH to age matched healthy controls to assess the differences related to LBD. Then, to test specific hypotheses related to MVH and CVH temporal phenomenology, we investigated the correlations between GMV, FA and MD with MVH and CVH temporal severity, duration and frequency.

## Methods

### Participants

We included both DLB and PDD participants as both are part of the LBD spectrum^24^. Twenty-eight LBD patients (16 with DLB and 12 with PDD) with a history of VH were recruited from the Department of Human Neuroscience of Sapienza University Hospital of Rome. Patients were considered trait hallucinators if they had experienced repeated hallucinations during the course of their illness. We set an arbitrary cut-off of having hallucinations in the preceding five years and a minimum of two separate hallucination episodes to define the hallucination trait. In practice, the average time since the last hallucination was 1.5 months for CVH and 3.3 months for MVH with a maximum of 18 months.

DLB diagnosis was made according to consensus criteria for probable DLB^25^; PDD diagnosis was made according to the Movement Disorder Society Clinical Diagnostic Criteria for Parkinson’s Disease^26^. Exclusion criteria for all participants were: MRI contraindications, a previous history of alcohol or substance abuse, significant neurological or psychiatric history, severe ocular diseases (e.g. glaucoma, macular degeneration), epilepsy, other forms of dementia, focal brain lesions on brain imaging, or the presence of other severe or unstable medical illnesses. Twenty Healthy controls (HC) matched for age and gender were also enrolled. The study was designed in accordance with the principles of the Declaration of Helsinki and was approved by the Ethical Committee of Sapienza University of Rome (Prot. 5178). Written informed consent was obtained from all individual participants included in the study.

All patients underwent a neurological examination including the Unified Parkinson’s Disease Rating Scale (UPDRS) part III^27^ and a structured clinical interview with caregivers to assess the presence of at least two of the core DLB clinical features: parkinsonism (i.e. hypokinesia, rest tremor, postural instability, rigidity), REM sleep behavioral disorders, cognitive fluctuations, and VH. All participants had normal or corrected to normal vision and a gross deficit in color perception was excluded by clinical screening in which patients were asked to indicate a specific color among many different color targets. Current treatment with levodopa, dopamine agonists (DA), antipsychotics, and acetyl-cholinesterase inhibitors (AchEI, e.g. rivastigmine) was also recorded. Levodopa and DA dosage were converted using the levodopa equivalent scale^28^, while antipsychotic drug dosage was converted using the chlorpromazine scale^29^. Global cognitive impairment was evaluated by means of Mini Mental State Examinations (MMSE)^30^. HC were included if they had a MMSE score > 27.

### VH assessment

A modified version of the North-East Visual Hallucinations Interview (NEVHI)^31^ was administered to all patients and their caregivers by experienced clinicians/researchers. The NEVHI is a semi-structured interview that assesses in detail the phenomenology of VH including its temporal aspects and time of last VH. The modified version included separate sections for simple, presence, illusion, complex, and passage hallucinations and other visual phenomena with questions about their frequency and time duration. The duration question asks “Approximately how long do these experiences usually last?” and the possible answers are “seconds”, “minutes”, “hours”, or “continuous”, scored respectively from 1 to 4. The frequency question asks “How often do they usually occur?” and the possible answers are: “less than every few months”, “every few months”, “every few weeks”, “every few days”, “every few hours”, “every few minutes”, “every few seconds”, or “continuously”, scored respectively from 1 to 8. The following temporal severity scores were derived:

MVH severity—the sum of duration X frequency for illusions, presence and passage and other (possible score 4–128).

MVH duration—the sum of duration for illusions, presence and passage and other (possible score 4–16).

MVH frequency—the sum of frequency for illusions, presence and passage and other (possible score 4–32).

CVH severity—duration X frequency for complex (possible score 1–32).

CVH duration—duration complex (possible score 1–4).

CVH frequency—frequency complex (possible score 1–8).

### MRI acquisition

Three‐dimensional, high‐resolution, T1‐weighted structural images were acquired for each participant (170 slices, in‐plane resolution = 192 × 256 mm, slice thickness = 1.2 mm, time repetition [TR] = 9 s, time echo [TE] = 4 ms), using a Siemens Magnetom Verio 3-Tesla scanner.

We also collected a diffusion-weighted image, acquired using Spin-Echo Planar Imaging (SE EPI; TR = 9.5 s, TE = 98 ms, matrix: 122 × 122, 65 slices, isotropic resolution = 2.1 mm^3^, b factor = 1000 s/mm^2^) with one image with no diffusion weighting (b0) and 64 images with diffusion gradients applied in 64 noncollinear directions.

### Statistical analyses

Statistical analyses of clinical scores were performed using SPSS (v. 27). Patients and HC were compared on demographics using an independent sample t-test. Spearman correlation coefficients were computed to assess the association between MVH and CVH scores and demographics (age and education), concomitant medications (levodopa, DA, AchEI, antipsychotics), MMSE, and parkinsonism severity (UPDRS) with significance threshold of *p* < 0.003 (Bonferroni corrected, based on 13 comparisons).

### VBM analysis

Images from 28 LBD patients and 20 healthy control (HC) subjects were included. We performed a VBM analysis on participants' T1-weighted structural images, using the Computational Anatomy Toolbox (CAT12), which runs within SPM12. The T1 anatomical images were manually checked for scanner artefacts and gross anatomical abnormalities. The images were then normalized using high-dimensional Diffeomorphic Anatomical Registration Through Exponentiated Lie Algebra (DARTEL) normalization and segmented into grey matter (GM), white matter, and cerebrospinal fluid (CSF). Segmented data were checked for quality and smoothed using an 8-mm full-width half-maximum (FWHM) kernel. Total intracranial volume (TIV) was estimated for each participant and used as a covariate at the second-level analyses.

Grey matter volume (GMV) was compared between patients and controls performing an independent sample t-test at the second-level. Multiple linear regressions analyses were further performed to investigate the association between the duration, frequency and severity of MVH and CVH and GMV in the patient group. Specifically, separate multiple regressions were performed to assess the impact of the duration, frequency and severity of VH, for both CVH and MVH. In each regression model, the opposite VH domain was entered as covariate of non-interest. MMSE and TIV were also entered as a covariates of non-interest in all multiple regression analyses.

### Diffusion data preprocessing

Twenty Seven LBD patients and 15 healthy control (HC) subjects were included in the Tract-Based Spatial Statistics (TBSS). One patient and 5 HC were excluded due to the presence of severe image artifacts.

DWI images were subjected to the following preprocessing pipeline^32^ using various tools, in the specified order:

- Thermal denoising was performed using the LPCA-Gaussian algorithm as implemented in DWIdenoisingPackage_r01^33^;
- Gibbs unringing^34^ was performed using MRtrix3's "mrdegibbs" (available at www.mrtrix.org);
- head motion and eddy current artifacts were corrected with outlier replacement^35^ using FSL's "eddy"^36^. The b matrices were rotated accordingly;
- field inhomogeneity correction was performed using "N4BiasFieldCorrection" from ANTs^37^ with the N4 algorithm^38^. A bias field correction was applied to the b0 image, and then the estimated field map was applied to all diffusion images.

After pre-processing the DWI data as specified, the diffusion tensor was estimated voxel-wise using the "dtifit" function from the FMRIB Software Library (FSL, https://fsl.fmrib.ox.ac.uk/fsl/fslwiki/FSL/). Fractional anisotropy (FA) and mean diffusivity (MD) were then derived for each subject. Next, voxel-wise group differences in FA and MD were investigated using the Tract-Based Spatial Statistics approach (TBSS)^39^, which allows for reliable alignment of fine white matter (WM) structures across subjects. In the TBSS analysis, FA maps were initially used to generate the WM skeleton. This was achieved by warping each subject's FA image into a common space using non-linear spatial transformation and averaging the normalized images to create a mean FA map. The mean FA map was subsequently thinned to create the WM skeleton, which represents the central portion of all WM tracts common to the groups. Subsequently, the FA and MD measures of each subject were projected onto the skeleton by searching perpendicular to the local skeleton structure for the maximum value. Voxel-wise group comparisons (LBD vs. HC) were then conducted on the skeletonized DTI measures (FA and MD). Additionally, correlation analyses were performed in the LBD patient group to investigate the relationship between each DTI measure (FA, MD) and clinical scores (duration, frequency and severity) related to minor (MVH) and complex hallucinations (CVH). In each correlation analysis, MMSE and the opposite VH domain score were included as nuisance variables to account for confounding factors. All statistical analyses, including group comparisons and correlations, were performed using the FSL tool "randomise"^40^, which performs permutation-based statistical analyses, with 2000 iterations. The resulting statistical maps were thresholded at p < 0.05, corrected for multiple comparisons using the Threshold-Free Cluster Enhancement (TFCE) method^41^ with 2D optimization. The anatomical location of significant clusters was determined and labelled using the JHU WM tractography atlas^42^.

## Results

Patients and HC did not differ for age, (t = − 0.099, *p* = 0.32), education (t = − 0.320, *p* = 0.75), and gender (χ2 = 0.421, *p* = 0.517). Participant’s demographics and clinical characteristics are shown in Table 1. All the patients had been taking their medication at a stable dosage for at least 12 months. No significant correlations were found between MVH indices and parkinsonism, MMSE, and concomitant medication, whereas a negative significant correlation was found between CVH severity and MMSE score (r = − 0.573, p_unc = 0.001). No correlations were found between MVH and CVH severity (r = 0.001 *p* = 0.99), duration (r = 0.64, *p* = 0.74) and frequency (r = 0.186, *p* = 0.34).

**Table 1:** Patient’s demographics and clinical characteristics.

Variable	Mean	SD
Diagnosis (DLB, number)	16	
Age	74.75 ±	5.76
Gender (male, number)	18	
education	9.63 ±	4.01
MMSE	20.96 ±	6.05
UPDRS	22.29 ±	12.32
CVH severity	6.89 ±	4.81
CVH duration	1.68 ±	0.98
CVH frequency	3.61 ±	1.77
MVH severity	5.43 ±	8.90
MVH duration	1.57 ±	2.00
MVH frequency	2.93 ±	3.04
Time from disease onset	4.54 ±	3.08
Antipsychotics chlopromazine equivalent dose	32.14 ±	55.21
Antipsychotics (number)	12	
AcheI	3.815 ±	4.501
AcheI (number)	13	
Levodopa equivalent dose	330.52 ±	374.72
Levodopa (number)	16	
Nevhi tot	12.32 ±	11.54

Supplementary Table 1 shows clinical characteristics separated for PDD and DLB patients.

**Figure 1:**
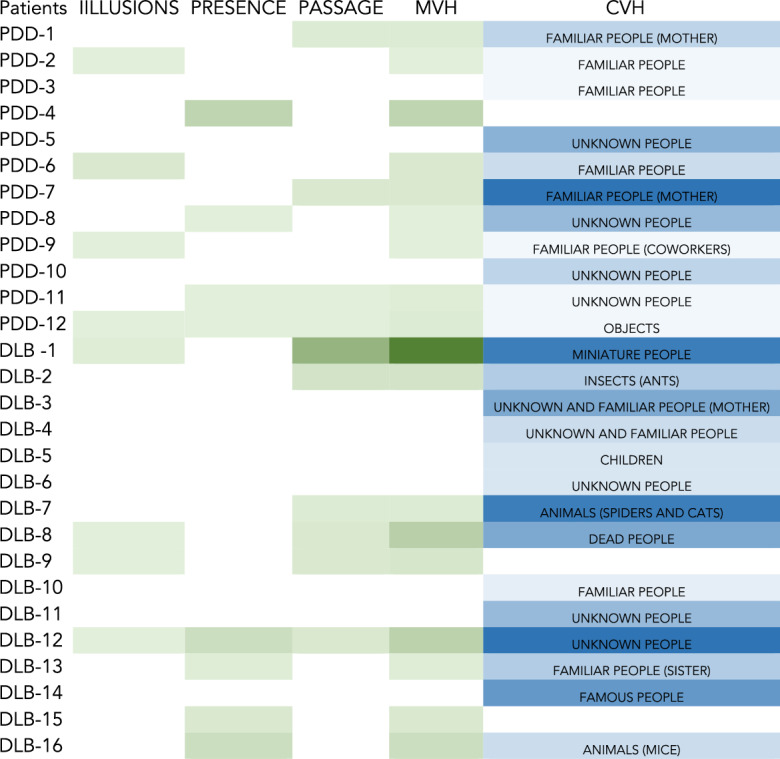
Colored patches of visual phenomena in LBD patients. Minor visual hallucinations, MVH light green-to-dark green; complex visual hallucinations, CVH light blue-to-dark blue represent the severity of symptoms reported by each patient for each phenomenon.

Among the LBD group, 18 patients had MVH, specifically 8 patients had illusions, 8 patients had presence hallucinations, 10 had passage hallucinations, 25 had complex hallucinations (3 patients had minor phenomena not associated with CVH). In 21 LBD patients CVH contents were familiar or unknown people. Figure 1 shows the patients’ VH phenomenology.

Twelve patients also had delusions, 5 of them had misidentification delusions, 5 misidentification delusions associated with paranoid delusions and 2 of them only paranoid delusions. Moreover 9 patients also had auditory hallucinations, 4 of these were associated with VH. Fourteen patients had mild depression, 8 patients also had agitation.

### VBM

Results of the independent sample t-test showed that patients had reduced GMV compared to controls in four clusters, corresponding to the bilateral fusiform gyrus (right: peak at MNI 36, − 33, − 20, t(1,45) = 6.22; left: peak at MNI − 27, − 4, − 39, t(1,45) = 5.86), to the left posterior cingulate cortex (peak at MNI − 2, − 38, 33, t(1,45) = 5.37) and the pars triangular of the inferior frontal gyrus in the right hemisphere (peak at MNI 50, 44, 4, t(1,45) = 5.32) (all pFWE < 0.05 at the cluster level, *p* uncorrected < 0.001 at the peak level) (Supplementary Fig. 1).

**Figure 2:**
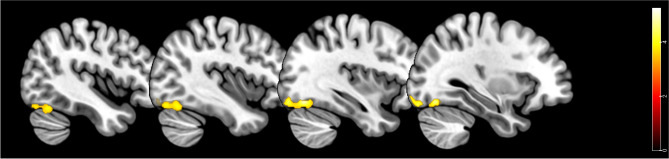
Clusters showing associations between reduced GMV and CVH duration in LBD patients (red to yellow patches show the T statistic of the regression model).

In the multiple regressions analyses, no significant association was found between GMV in the patient group and MVH duration, frequency or severity. There was no significant association between CVH frequency and severity and GMV. The duration of CVH was negatively associated with GMV in one cluster in the left fusiform gyrus (peak at MNI: − 34, − 72, 15, t(1,23) = 5.35, pFWE < 0.05 at the cluster level, p uncorrected < 0.001 at the peak level) (Fig. 2).

### Fractional anisotropy

The results of the TBSS analysis revealed widespread decreased fractional anisotropy (FA) in LBD patients compared to HC subjects (HC > LBD), showing significant clusters of lower FA in patients in the bilateral anterior thalamic radiation, corticospinal tract, superior and inferior parts of left cingulum and forceps minor and major, as well as in bilateral inferior fronto-occipital fasciculi, inferior and superior longitudinal fasciculi (in the temporal portion) and uncinate fasciculus (Supplementary Fig. 2). No significant suprathreshold voxel was detected for the opposite contrast.

No significant correlations were found between FA and MVH or CVH indices.

### Mean diffusivity

TBSS analysis revealed a widespread increased mean diffusivity (MD) in LBD patients compared to HC (LBD > HC). We found significant clusters of increased MD in patients in the bilateral anterior thalamic radiation, corticospinal tract, superior and inferior parts of cingulum and forceps minor and major, as well as in bilateral inferior fronto-occipital fasciculi, inferior and superior longitudinal fasciculi (in the temporal portion) and uncinate fasciculus (Supplementary Fig. 3). No significant suprathreshold voxel was detected for the opposite contrast.

**Figure 3:**
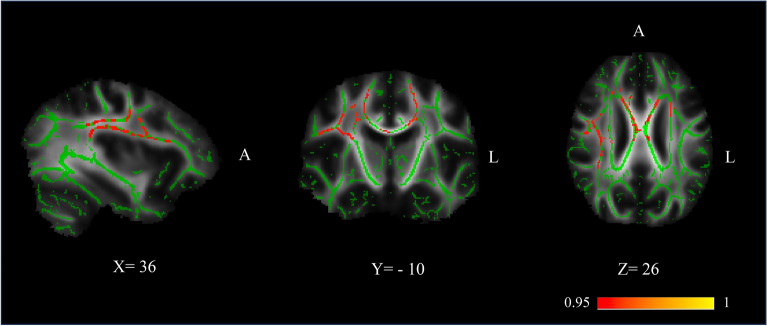
TBSS correlation between mean diffusivity (MD) and MVH severity in LBD. Mean fractional anisotropy skeleton (in green) overlaid on the mean fractional anisotropy map. Voxels that showed value of mean diffusivity significantly negatively correlated with MVH severity are overlaid in red-to-yellow patches, 1-p for convenience of display; thus, thresholding at 0.95 gives significant clusters (see also https://fsl.fmrib.ox.ac.uk/fsl/fslwiki/TBSS). *Notes*: A = anterior; L = left.

In LBD patients, significant negative correlations were found between MD and MVH severity, including the bilateral anterior thalamic radiation, corticospinal tract, superior cingulum, forceps minor, as well as in bilateral inferior fronto-occipital fasciculi, superior longitudinal fasciculi (including the left temporal portion), right inferior longitudinal fasciculus and left uncinate fasciculus (Fig. 3). There was no significant correlation between MD and CVH indices, or between MD and MVH frequency and duration.

## Discussion

In this study we investigated grey and white matter alterations associated with MVH and CVH temporal phenomenology in LBD patients.

### Topological—grey matter findings

We found decreased GM volume bilaterally in the fusiform gyrus (FG), in the left posterior cingulate and in the right inferior frontal gyrus in LBD with VH compared to HC. Although the LBD versus HC comparison is not specific to VH and captures differences due to LBD overall, the regions identified have been implicated in VH in previous studies^15,43,44^ suggesting they may represent structural hallmarks of functional alterations in networks contributing to VH. The involvement of the fusiform gyrus, part of the ventral visual stream, may account for the typical VH contents found in LBD and has already been reported in VBM studies of VH in the LBD spectrum^16,43^. The alteration in the left posterior cingulate, part of the default mode network (DMN), may also contribute to VH occurrence. For example, an fMRI study in PD patients with VH found increased engagement of the DMN^45^ and decreased connectivity of the DMN was found to be associated with CVH frequency in LBD^9^. The inferior frontal gyrus (IFG) is a region that preferentially contributes to psychotic symptoms in schizophrenia^46^ and may contribute to the loss of inhibitory control^47^. Thus, it might be possible that cortical volume loss in these regions contribute to VH as emerged from previous studies on VH in LBD^14,48^. Frontal volume loss may sustain an attention deficit contributing to VH when associated with visuoperceptual impairment, according to the Perception and Attention deficit model^49^. Moreover, the IFG can be considered part of the ventral attention network (VAN) and dysfunction in this area might be interpreted in light of Shine’s model stating that an increased engagement of the VAN contributes to VH occurrence^50^. Frontal regions are also implicated in reality monitoring processes. A deficit in this process may lead to internal representations experienced as real perceptual experiences^51^.

Supporting our MVH hypothesis, we did not find any associations between GM and MVH phenomenology suggesting MVH phenomena are not associated with grey matter alterations per se but rather to functional alterations in the connectivity of specific visual networks (see below). Consistent with our CVH hypothesis we found that CVH duration was negatively associated with GM volume in the left FG. Neurodegenerative changes in the FG may lead to a spontaneous activation of this region that sustains CVH duration, the functional specialisations of the region accounting for the characteristic content of CVH^9^. The finding is consistent with previous structural^16,43^ and functional MRI studies^9,52,53^ that reported an association between fusiform gyrus alterations and VH. The fusiform gyrus contains regions specialized for face perception and activity in the fusiform gyrus and other specialised visual cortices has been associated with hallucinations of familiar people in schizophrenia^54^. Although most of the patients experienced CVH of people with faces rather than disembodied faces alone, it seems plausible the specialisation of this area of GM volume loss is linked to the contents of the CVH reported. The association between GMV and CVH duration but not frequency is consistent with previous evidence that suggests that the two temporal aspects of phenomenology relate to different mechanisms^9^.

### Hodological–white matter findings

TBSS analysis revealed widespread damage of white matter bundles in LBD patients compared to HC subjects, confirming previous findings about alterations of structural connectivity in DLB patients. These results are not specific to VH and are thus likely to reflect the axonal damage typical of the LBD neuropathological process. Indeed, our findings are in line with those from previous studies showing diffuse alterations of white matter in these patients^55^, especially prominent in posterior regions^56^.

Although we predicted MVH would be linked to changes in specific networks, unexpectedly, we found widespread regions of negative correlation between MD and MVH severity. These regions included the bilateral anterior thalamic radiation, corticospinal tract, superior cingulum, forceps minor, as well as the bilateral inferior fronto-occipital fasciculi, superior longitudinal fasciculi, right inferior longitudinal fasciculus and left uncinate fasciculus. The widespread distribution of the findings suggests that MVH are associated with the integrity of white matter bundles across several functional networks. We note that the association found was specific to MD and that we did not find any association with FA. One possible explanation is that FA might be a less sensitive measure compared to MD when it comes to brain tissue changes. Both FA and MD are influenced by various factors, including axonal count and density, degree of myelination, and fibers organization, such as the presence of crossing fibers. However, unlike MD, there are certain types of brain tissue damage that may not result in changes in FA or may cause an increase or decrease in FA. For instance, a selective loss of unidirectional fibers in a brain area with crossing fibers can lead to an increase in FA, while the loss of fibers in a white matter region characterized by only one direction (such as the corpus callosum) can result in a decrease in FA^57,58^. MD, which measures the overall diffusivity of water molecules in tissue, better reflects microstructural changes in white matter and is a better indicator of brain tissue damage. An increase in MD is usually associated with the presence of damage such as axonal loss, demyelination, or inflammation. It is likely that MD captures the fine-grained variations associated with clinical indices more effectively than FA in this patient group.

**Figure 4:**
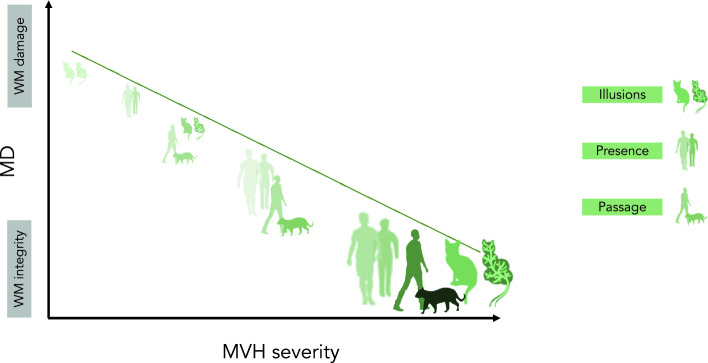
A figurative graph of the negative correlation between Mean diffusivity (with implied WM integrity) and MVH severity. MVH are shown in a color-scale of light green-to-dark green representing the severity of illusions, presence and passage hallucinations.

An important observation is that the direction of association between MVH and MD was negative. Although counterintuitive, we may speculate that the networks underlying MVH need to be partly intact and functional for MVH experiences to occur. As widespread alteration of structural connectivity disrupts the networks, the MVH become less frequent and have shorter duration (a reduction in MVH temporal severity) (see Fig. 4). Thus, in contrast to CVH, MVH can be considered closer to normal perceptual illusion phenomena (elicited from external stimuli but misperceived) and require intact or minimally impaired visual perceptual systems to occur.

MVH include a range of phenomena (passage, presence as well as illusions) that do not necessarily share the same mechanism but often occur in the same patient at different times. Passage hallucinations have been hypothesized to be due to a dysfunction between brainstem eye movement control regions, and subcortical and cortical motion pathways, including dorsal visual stream areas^1^. Presence hallucinations, described as the feeling that someone is close by or has just left the room, have been interpreted as a form of palinopsia relying on dorsal visual stream involvement^1^ and recent evidence using a robotic stimulation paradigm suggests they result from sensorimotor conflict^59,60^.

It is possible that MVH collectively, both when elicited from an external stimulus (illusions) or when internally produced or through sensorimotor conflict (passage and presence) need the integrity of fibers that connect the ventral visual stream, the dorsal attention network and the dorsal visual stream. The Superior longitudinal fasciculus is a white matter bundle connecting the frontal and parietal lobes^61^. It connects part of the dorsal attention network (DAN), and the ventral attention network (VAN)^62^. The frontoparietal network plays a role in the top-down control of spatial attention^63,64^. Overall, the integrity of these tracts may be required for the attentional control processes associated with MVH to function. This hypothesis is also in line with a recent fMRI study in PD patients with VH who showed increased top-down effective connectivity from the left prefrontal cortex to primary visual cortex compared to PD without VH. This pattern of connectivity was also reported to be predictive of VH occurrence and associated with their severity^65^.

Other pathways implicated in our findings may have specific roles in subcategories of MVH. The inferior fronto-occipital fasciculus has a role in the visual recognition system^66^ and underlies the integration of complex movements, perception, and location of body parts between sensory-motor, occipital areas, and ventro-lateral prefrontal cortex. It is involved in object identification, visual attention, and planning of visually guided movements^67^. It is possible that the integrity of these tracts might be specifically related to passage hallucinations. The uncinate fasciculus connects face processing areas and the limbic regions responsible for emotional attribution of facial percepts and has been implicated in Capgras syndrome^68,69^ which, although not present in our patient cohort, occurs in LBD^1^. Its preservation might be why there is an absence of emotional dysfunction in MVH. The forceps minor, the superior cingulate and the anterior thalamic radiation are part of the fronto-thalamic loop^70^ that is involved in executive functions^71,72^ which have been linked to VH in PD (see ffytche et al.^1^). The inferior longitudinal fasciculus is an associative bundle transmitting visual information from occipital areas to the temporal lobe and is involved in visual object recognition and implicated in a several visual perceptual disorders^73,74^. The cortico-spinal tract integrity might be consistent with the hypothesis that presence hallucinations can be interpreted as an alteration of self-related sensorimotor processing and this hypothesis has been supported by studies that induced presence hallucinations using a robotic sensorimotor paradigm^59,60^.

As noted above, the negative correlation with MD suggests that the integrity of connectivity among these networks is a pre-condition to allow MVH to occur. In other words, MVH may implicate dysfunction or slowing of visual processing but, at the same time, the processing areas have to be structurally connected in order for an MVH to occur. Although different networks may link to different symptoms within the MVH category, a key role might be played by top-down influences on visual processing and attentional modulation between DAN, VAN and DMN. These findings suggest that, for MVH to occur, the visual system needs to be functioning, even if in a pathological way. This might explain why MVH more often occur in early LBD disease stages and are more frequent in PD, where alterations of structural connectivity among cortical areas are not expected to be severe. Taken together with the lack of association between GMV alterations and MVH temporal severity and the lack of correlation with cognitive impairment, our findings lend further support to the view that MVH are typically related to changes occurring in early LBD, and depend on a different mechanism compared to CVH^1,9^. CVH seem to be internally generated, likely as the consequence of an aberrant activation of specialized visual regions due to GM damage, and can occur without the integrity of visual and attentional networks.

Overall, the alterations of brain regions and networks may represent the predisposing preconditions for VH in LBD. From a hodotopic perspective, our findings support the idea that VH phenomenology may help identify brain regions and networks underlying VH and better understand dysfunction of cortical regions/networks occurring during the VH state^8^.

The study has some limitations. First, the sample did not include LBD patients without VH to disentangle effects related to disease and effects related to VH in the comparison with healthy controls. Moreover, we could not investigate the structural alterations associated with each subcategory of CVH and MVH (e.g. presence versus illusion). Future studies including LBD patients experiencing only one type of phenomenon could help to shed light on the neural basis of individual symptoms. The DTI images were analysed using a TBSS approach but a probabilistic approach may better capture changes in white matter bundles associated with VH. Recent probabilistic tracking studies have investigated white matter alterations associated with cholinergic pathways^75^ and this approach might help identify which neurotransmitter system is most closely associated with VH phenomenology. Furthermore, TBSS did not allow us to investigate connectivity with subcortical regions which might play a role in VH pathophysiology in LBD. Future studies including more advanced neuroimaging approaches to investigate structural connectivity are required.

This study provides evidence that MVH and CVH are underpinned by a complex pattern of white and grey matter changes in LBD, supporting the hypothesis that VH phenomenology in LBD may mirror neuropathological progression^1^. Moreover, these findings further support the hodotopic hypothesis^8^ that an understanding of hallucinations requires both consideration of alterations in white matter network connectivity and localised grey matter changes. Our findings may be considered a first step in identifying brain networks that need to be intact or minimally dysfunctional for specific hallucination subtypes to occur.

### Supplementary Information

Supplementary Figure 1.

Supplementary Figure 2.

Supplementary Figure 3.

Supplementary Information 4.

Supplementary Legends.

## Data Availability

The datasets generated during and/or analysed during the current study are available from the corresponding author on reasonable request.

## References

[1] Ffytche DH (2017). The psychosis spectrum in Parkinson disease. Nat. Rev. Neurol..

[2] Lenka A, Pagonabarraga J, Pal PK, Bejr-Kasem H, Kulisvesky J (2019). Minor hallucinations in Parkinson disease: A subtle symptom with major clinical implications. Neurology.

[3] Onofrj M (2013). Visual hallucinations in PD and Lewy body dementias: Old and new hypotheses. Behav. Neurol..

[4] Shine JM, Halliday GM, Naismith SL, Lewis SJG (2011). Visual misperceptions and hallucinations in Parkinson’s disease: Dysfunction of attentional control networks?. Mov. Disord..

[5] Diederich NJ, Goetz CG, Stebbins GT (2005). Repeated visual hallucinations in Parkinson’s disease as disturbed external/internal perceptions: Focused review and a new integrative model. Mov. Disord..

[6] Friston KJ (2005). Hallucinations and perceptual inference. Behav. Brain Sci..

[7] Collerton D (2023). Understanding visual hallucinations: A new synthesis. Neurosci Biobehav. Rev..

[8] Ffytche DH (2008). The hodology of hallucinations. Cortex.

[9] D’Antonio F (2022). Visual hallucinations in Lewy body disease: Pathophysiological insights from phenomenology. J. Neurol..

[10] Cheng HC, Ulane CM, Burke RE (2010). Clinical progression in Parkinson disease and the neurobiology of axons. Ann. Neurol..

[11] Tagliaferro P, Burke RE (2016). Retrograde axonal degeneration in Parkinson disease. J. Parkinsons Dis..

[12] Caminiti SP (2017). Axonal damage and loss of connectivity in nigrostriatal and mesolimbic dopamine pathways in early Parkinson’s disease. Neuroimage Clin..

[13] Fathy YY (2022). Axonal degeneration in the anterior insular cortex is associated with Alzheimer’s co-pathology in Parkinson’s disease and dementia with Lewy bodies. Transl. Neurodegener..

[14] Pezzoli S, Cagnin A, Bandmann O, Venneri A (2017). Structural and functional neuroimaging of visual hallucinations in lewy body disease: A systematic literature review. Brain Sci..

[15] Pezzoli S (2021). Neuroanatomical and cognitive correlates of visual hallucinations in Parkinson’s disease and dementia with Lewy bodies: Voxel-based morphometry and neuropsychological meta-analysis. Neurosci. Biobehav. Rev..

[16] Goldman JG (2014). Visuoperceptive region atrophy independent of cognitive status in patients with Parkinson’s disease with hallucinations. Brain.

[17] Janzen J (2012). The pedunculopontine nucleus is related to visual hallucinations in Parkinson’s disease: preliminary results of a voxel-based morphometry study. J. Neurol..

[18] Weil RS, Hsu JK, Darby RR, Soussand L, Fox MD (2019). Neuroimaging in Parkinson’s disease dementia: connecting the dots. Brain Commun..

[19] Pagonabarraga J (2014). Neural correlates of minor hallucinations in non-demented patients with Parkinson’s disease. Parkinsonism Relat. Disord..

[20] Bejr-kasem H (2021). Minor hallucinations reflect early gray matter loss and predict subjective cognitive decline in Parkinson’s disease. Eur. J. Neurol..

[21] Zorzi G (2021). White matter abnormalities of right hemisphere attention networks contribute to visual hallucinations in dementia with Lewy bodies. Cortex.

[22] Mehraram R (2022). Functional and structural brain network correlates of visual hallucinations in Lewy body dementia. Brain.

[23] Delli Pizzi S (2014). Relevance of subcortical visual pathways disruption to visual symptoms in dementia with Lewy bodies. Cortex.

[24] Jellinger KA, Korczyn AD (2018). Are dementia with Lewy bodies and Parkinson’s disease dementia the same disease?. BMC Med..

[25] Mckeith, I. G. et al.* Diagnosis and Management of Dementia with Lewy Bodies Fourth Consensus Report of the DLB Consortium*. (2017). 10.1212/WNL.0000000000004919 29438029

[26] Postuma RB (2015). MDS clinical diagnostic criteria for Parkinson’s disease. Mov. Disord..

[27] Goetz CG (2008). Movement disorder society-sponsored revision of the unified parkinson’s disease rating scale (MDS-UPDRS): Scale presentation and clinimetric testing results. Mov. Disord..

[28] Möller JC (2005). Pharmacotherapy of Parkinson’s disease in Germany. J. Neurol..

[29] Woods SW (2003). Chlorpromazine equivalent doses for the newer atypical antipsychotics. J. Clin. Psychiatry.

[30] Folstein MF, Folstein SE, Mchugh PR (1975). ‘Mini-mental state’ a practical method for grading the cognitive state of patients for the clinician. J. Psychiatr. Res..

[31] Mosimann UP (2008). A semi-structured interview to assess visual hallucinations in older people. Int. J. Geriatr. Psychiatry.

[32] Maximov II, Alnæs D, Westlye LT (2019). Towards an optimised processing pipeline for diffusion magnetic resonance imaging data: Effects of artefact corrections on diffusion metrics and their age associations in UK Biobank. Hum. Brain Mapp..

[33] Manjón JV (2013). Diffusion weighted image denoising using overcomplete local PCA. PLoS One.

[34] Kellner E, Dhital B, Kiselev VG, Reisert M (2016). Gibbs-ringing artifact removal based on local subvoxel-shifts. Magn. Reson. Med..

[35] Andersson JLR, Graham MS, Zsoldos E, Sotiropoulos SN (2016). Incorporating outlier detection and replacement into a non-parametric framework for movement and distortion correction of diffusion MR images. Neuroimage.

[36] Andersson JLR, Sotiropoulos SN (2016). An integrated approach to correction for off-resonance effects and subject movement in diffusion MR imaging. Neuroimage.

[37] Avants BB (2011). A reproducible evaluation of ANTs similarity metric performance in brain image registration. Neuroimage.

[38] Tustison NJ (2010). N4ITK: Improved N3 bias correction. IEEE Trans. Med. Imaging.

[39] Smith SM (2006). Tract-based spatial statistics: Voxelwise analysis of multi-subject diffusion data. Neuroimage.

[40] Winkler AM, Ridgway GR, Webster MA, Smith SM, Nichols TE (2014). Permutation inference for the general linear model. Neuroimage.

[41] Smith SM, Nichols TE (2009). Threshold-free cluster enhancement: Addressing problems of smoothing, threshold dependence and localisation in cluster inference. Neuroimage.

[42] Mori S, Wakana S, Nagae-Poetscher LM, van Zijl PCM (2005). MRI atlas of human white matter. AJNR Am. J. Neuroradiol..

[43] Bejr-kasem H (2019). Disruption of the default mode network and its intrinsic functional connectivity underlies minor hallucinations in Parkinson’s disease. Mov. Disord..

[44] Pezzoli S, Cagnin A, Antonini A, Venneri A (2019). Frontal and subcortical contribution to visual hallucinations in dementia with Lewy bodies and Parkinson’s disease. Postgrad. Med..

[45] Shine JM (2014). The role of dysfunctional attentional control networks in visual misperceptions in Parkinson’s disease. Hum. Brain Mapp..

[46] Iwashiro N (2012). Localized gray matter volume reductions in the pars triangularis of the inferior frontal gyrus in individuals at clinical high-risk for psychosis and first episode for schizophrenia. Schizophr. Res..

[47] Boen R, Raud L, Huster RJ (2022). Inhibitory control and the structural parcelation of the right inferior frontal gyrus. Front. Hum. Neurosci..

[48] Sanchez-Castaneda C (2010). Frontal and associative visual areas related to visual hallucinations in dementia with Lewy bodies and Parkinson’s disease with dementia. Mov. Disord..

[49] Collerton D, Perry E, McKeith I (2005). Why people see things that are not there: A novel perception and attention deficit model for recurrent complex visual hallucinations. Behav. Brain Sci..

[50] Shine JM, O’Callaghan C, Halliday GM, Lewis SJG (2014). Tricks of the mind: Visual hallucinations as disorders of attention. Prog. Neurobiol..

[51] Pagonabarraga J, Bejr-Kasem H, Martinez-Horta S, Kulisevsky J (2024). Parkinson disease psychosis: From phenomenology to neurobiological mechanisms. Nat. Rev. Neurol..

[52] Yao N (2015). Resting activity in visual and corticostriatal pathways in Parkinson’s disease with hallucinations. Parkinsonism Relat. Disord..

[53] Hepp DH (2017). Loss of functional connectivity in patients with Parkinson disease and visual hallucinations. Radiology.

[54] Oertel V (2007). Visual hallucinations in schizophrenia investigated with functional magnetic resonance imaging. Psychiatry Res. Neuroimaging.

[55] Delli Pizzi S (2015). Structural connectivity is differently altered in dementia with Lewy body and Alzheimer’s disease. Front. Aging Neurosci..

[56] Nedelska Z (2015). White matter integrity in dementia with Lewy bodies: A voxel-based analysis of diffusion tensor imaging. Neurobiol. Aging.

[57] Winston GP (2012). The physical and biological basis of quantitative parameters derived from diffusion MRI. Quant. Imaging Med. Surg..

[58] Figley CR (2022). Potential pitfalls of using fractional anisotropy, axial diffusivity, and radial diffusivity as biomarkers of cerebral white matter microstructure. Front. Neurosci..

[59] Bernasconi F (2021). Robot-induced hallucinations in Parkinson’s disease depend on altered sensorimotor processing in fronto-temporal network. Sci. Transl. Med..

[60] Blanke O, Bernasconi F, Potheegadoo J (2023). Phantom boarder relates to experimentally-induced presence hallucinations in Parkinson’s disease. Mov. Disord. Clin. Pract..

[61] de Thiebaut Schotten M (2011). Atlasing location, asymmetry and inter-subject variability of white matter tracts in the human brain with MR diffusion tractography. Neuroimage.

[62] de Thiebaut Schotten M, Dell’Acqua F, Valabregue R, Catani M (2012). Monkey to human comparative anatomy of the frontal lobe association tracts. Cortex.

[63] Gazzaley A, Nobre AC (2012). Top-down modulation: Bridging selective attention and working memory. Trends Cogn. Sci..

[64] Nobre AC, Mesulam MM (2014). Large-Scale Networks for Attentional Biases.

[65] Thomas GEC (2023). Changes in both top-down and bottom-up effective connectivity drive visual hallucinations in Parkinson’s disease. Brain Commun..

[66] Conner AK (2018). A connectomic atlas of the human cerebrum-chapter 13: Tractographic description of the inferior fronto-occipital fasciculus. Oper. Neurosurg..

[67] de Benedictis A, Marras CE, Laurent PETIT, Sarubbo S (2021). The inferior fronto-occipital fascicle: a century of controversies from anatomy theaters to operative neurosurgery. J. Neurosurg. Sci..

[68] Hirstein W, Ramachandran VS (1997). Capgras syndrome : A novel probe for understanding the neural representation of the identity and familiarity of persons. Proc. R. Soc. Lond. Ser. B Biol. Sci..

[69] Von Der Heide RJ, Skipper LM, Klobusicky E, Olson IR (2013). Dissecting the uncinate fasciculus: Disorders, controversies and a hypothesis. Brain.

[70] Catani M, de Schotten MT (2008). A diffusion tensor imaging tractography atlas for virtual in vivo dissections. Cortex.

[71] De Bourbon-Teles J (2014). Thalamic control of human attention driven by memory and learning. Curr. Biol..

[72] Mamiya PC, Richards TL, Kuhl PK (2018). Right forceps minor and anterior thalamic radiation predict executive function skills in young bilingual adults. Front. Psychol..

[73] Ffytche DH, Blom JD, Catani M (2010). Disorders of visual perception. J. Neurol. Neurosurg. Psychiatry.

[74] Catani M (2012). Beyond cortical localization in clinico-anatomical correlation. Cortex.

[75] Schumacher J (2022). Cholinergic white matter pathways in dementia with Lewy bodies and Alzheimer’s disease. Brain.

