# Supplementary figures and images for: Distinct grey and white matter changes are associated with the phenomenology of visual hallucinations in Lewy Body Disease
Source: Sci Rep. 2024 Jun 26;14:14748. doi: 10.1038/s41598-024-65536-w (PMC11208453; doi:10.1038/s41598-024-65536-w)

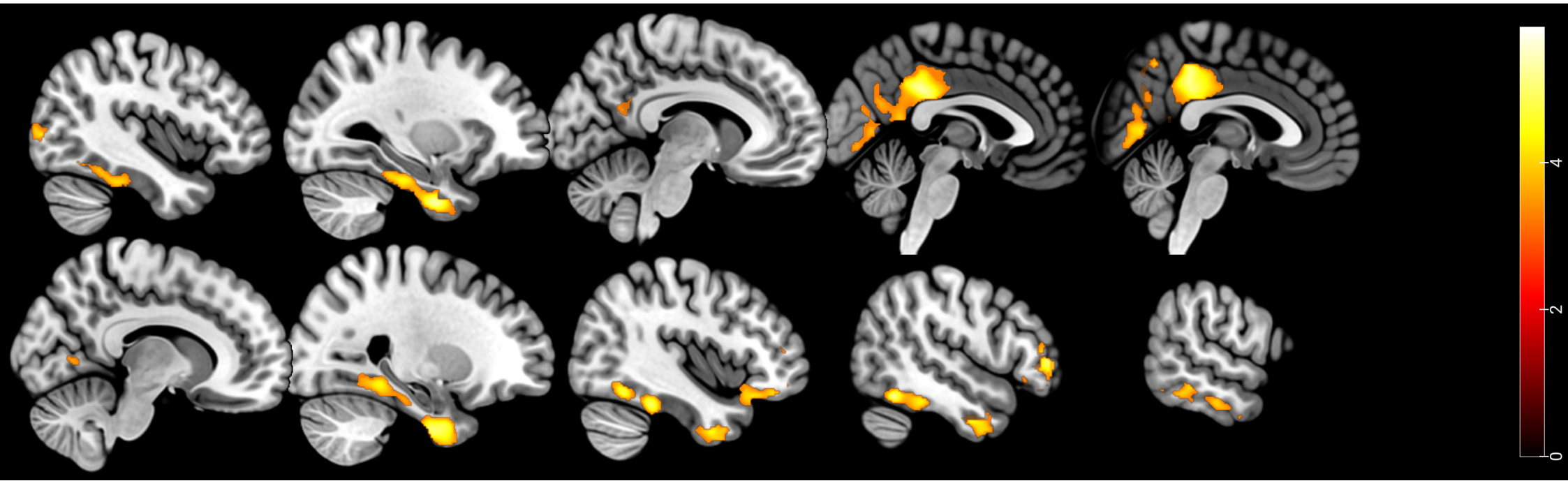

Supplement: Supplementary file 1 — Supplementary Figure 1. [file 41598_2024_65536_MOESM1_ESM.pdf]

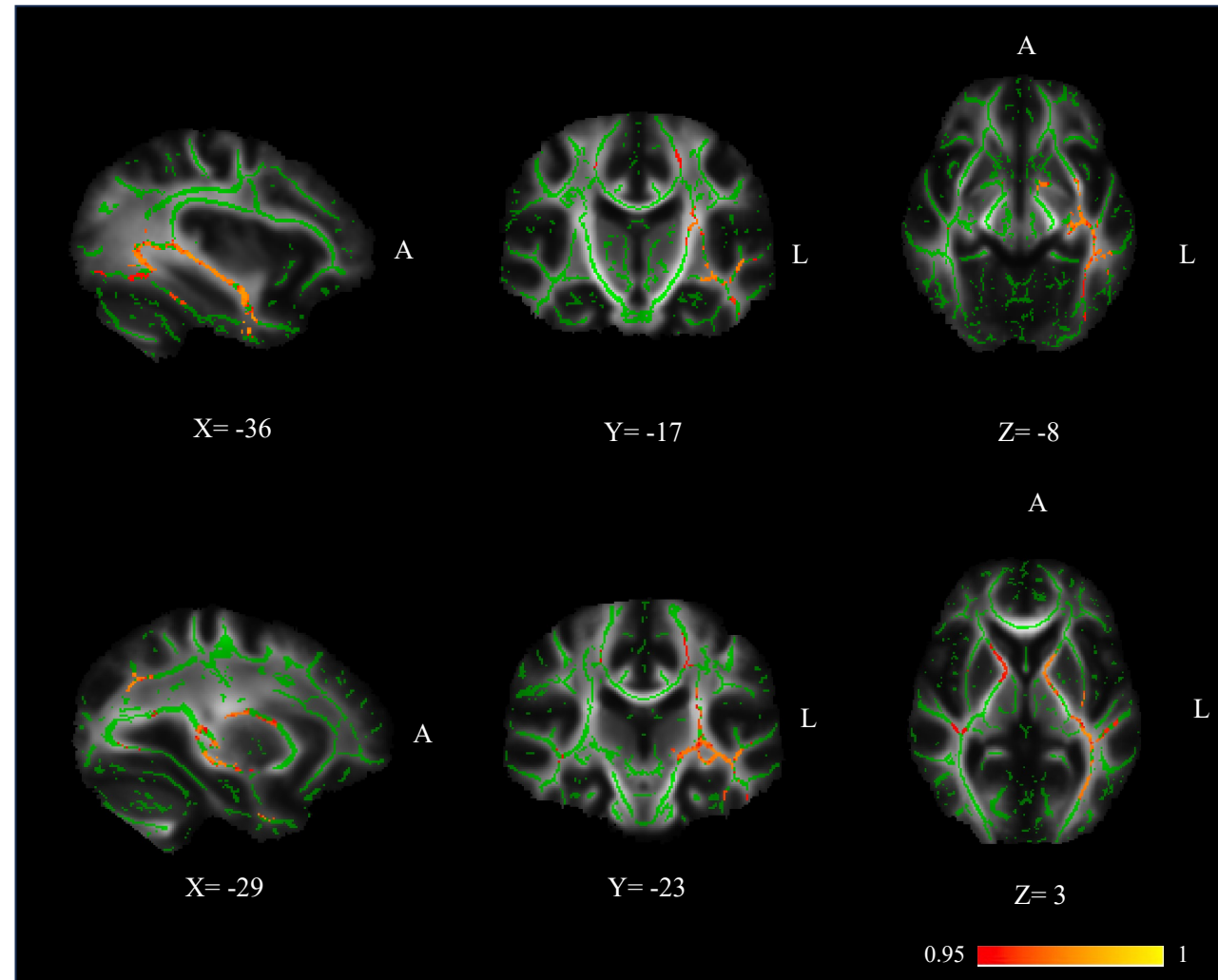

Supplement: Supplementary file 2 — Supplementary Figure 2. [file 41598_2024_65536_MOESM2_ESM.pdf]

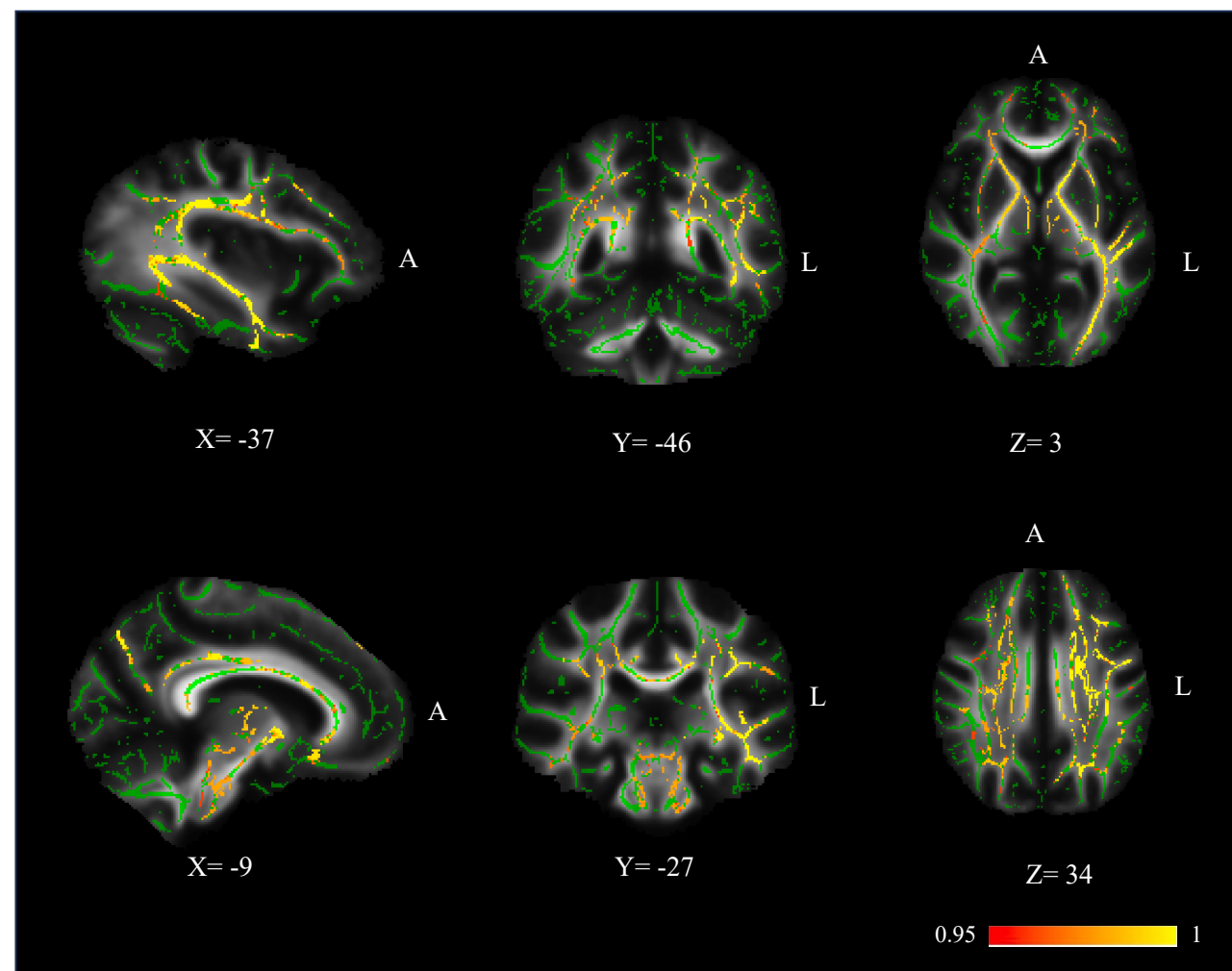

Supplement: Supplementary file 3 — Supplementary Figure 3. [file 41598_2024_65536_MOESM3_ESM.pdf]
