# Supplementary material for: Distinct grey and white matter changes are associated with the phenomenology of visual hallucinations in Lewy Body Disease
Source: Sci Rep. 2024 Jun 26;14:14748. doi: 10.1038/s41598-024-65536-w (PMC11208453; doi:10.1038/s41598-024-65536-w)
Supplement: Supplementary file 4 — Supplementary Information 4. [file 41598_2024_65536_MOESM4_ESM.pdf]

Supplementary materials

Supplementary Table 1

Variable	DLB (16)	PDD (12)
Age (mean ±sd)	74,8 ±6,09	74,5 ± 5,5
Gender (male, number)	7	11
Education (mean ±sd)	8,07±3,8	11,5± 3,5
MMSE (mean ±sd)	19,3±6,1	23,1± 5,3
RBD (number)	13	9
Fluctuations (number)	10	3
UPDRS (mean ±sd)	20,3±10,7	24,9± 14,1
CVH severity (mean ±sd)	7,1 ±5,3	6,5 ± 4,2
CVH duration (mean ±sd)	1,7 ±0,9	1,6 ± 1,07
CVH frequency (mean ±sd)	3,5±2	3,6± 1,4
MVH severity (mean ±sd)	6,3±11,4	4,1± 3,7
MVH duration (mean ±sd)	1,7 ± 2,5	1,3± 1,07
MVH frequency (mean ±sd)	3± 3,6	2,8± 2,03
NEVHI total	13,5 ± 14,8	10,7± 4,8
NPI (mean ±sd)	28,4 ± 18	30,6± 15,7
Months between PD onset and dementia onset (mean ±sd)	n.a.	61,8± 17,5
Time from dementia onset (mean ±sd)	3,8±1,7	5,5 ± 4,1
Antipsychotics chlopromazine equivalent dose	37,5 ± 54	25± 58,3
Antipsychotics (number)	9	3
AcheI dose	5,2 ±4,5	1,7 ± 3,8
AcheI (number)	10	3
Levodopa equivalent dose	94,6 ±171	673,6± 320
Levodopa (number)	6	10

Supplementary analyses

Since CVH and MMSE were correlated, we investigated whether there is a difference in MMSE score in patients with severe CVH compared to with mild CVH. We divided the patients on basis of CVH severity median score (6) and we compared the two groups (low severity hallucinators vs high severity hallucinators) to test differences in MMSE scores.
The Mann Whitney test did not reveal any significant difference between the groups (U=51.5, p=0.147).

	Mild CVH (n=12)	Severe CVH (n=13)	U- Mann Whitney	p-value
MMSE	21.8 ± 5.7	18.6 ± 5.8	51.5	0.147

We performed a Mann Whitney test to verify whether PDD and DLB differed in MVH and CVH severity, but no significant differences emerged either for MVH (U=88, p=0.703) or CVH severity (U=94.5, p= 0,94).

	PDD (n=12)	DLB (n=16)	U- Mann Whitney	p-value
MVH severity	4.1± 3.7	6.3±11.4	88	0.703
CVH severity	7.1 ±5.3	6.5 ± 4.2	94.5	0.94
