# Supplementary material for: Distinct grey and white matter changes are associated with the phenomenology of visual hallucinations in Lewy Body Disease
Source: Sci Rep. 2024 Jun 26;14:14748. doi: 10.1038/s41598-024-65536-w (PMC11208453; doi:10.1038/s41598-024-65536-w)
Supplement: Supplementary file 5 — Supplementary Legends. [file 41598_2024_65536_MOESM5_ESM.docx]

**Supplementary Figure 1**. Red to yellow patches show the T statistic of the comparison between GM volume in LBD and HC (p-FDR < 0.05 at the cluster level and p < 0.001 uncorrected at the peak level).

**Supplementary Figure 2** Results of the comparison of FA between LBD patients and HC subjects with TBSS analysis. Mean fractional anisotropy skeleton (in green) overlaid on the mean fractional anisotropy map. Voxels that value of fractional anisotropy significantly differed between HC and LBD are overlaid in red-to-yellow patches, 1-p for convenience of display; thus, thresholding at 0.95 gives significant clusters (see also https://fsl.fmrib.ox.ac.uk/fsl/fslwiki/TBSS). Notes: A = anterior; L = left.

**Supplementary Figure 3** Results of the comparison of MD between LBD patients and HC subjects with TBSS analysis. Mean fractional anisotropy skeleton (in green) overlaid on the mean fractional anisotropy map. Voxels that value of mean diffusivity significantly differed between HC and LBD are overlaid in red-to-yellow patches, 1-p for convenience of display; thus, thresholding at 0.95 gives significant clusters (see also https://fsl.fmrib.ox.ac.uk/fsl/fslwiki/TBSS). Notes: A = anterior; L = left.
